# Supplementary figures and images for: Effect of a multi-faceted rapid response system re-design on repeat calling of the rapid response team
Source: PLoS One. 2022 Mar 24;17(3):e0265485. doi: 10.1371/journal.pone.0265485 (PMC8947019; doi:10.1371/journal.pone.0265485)

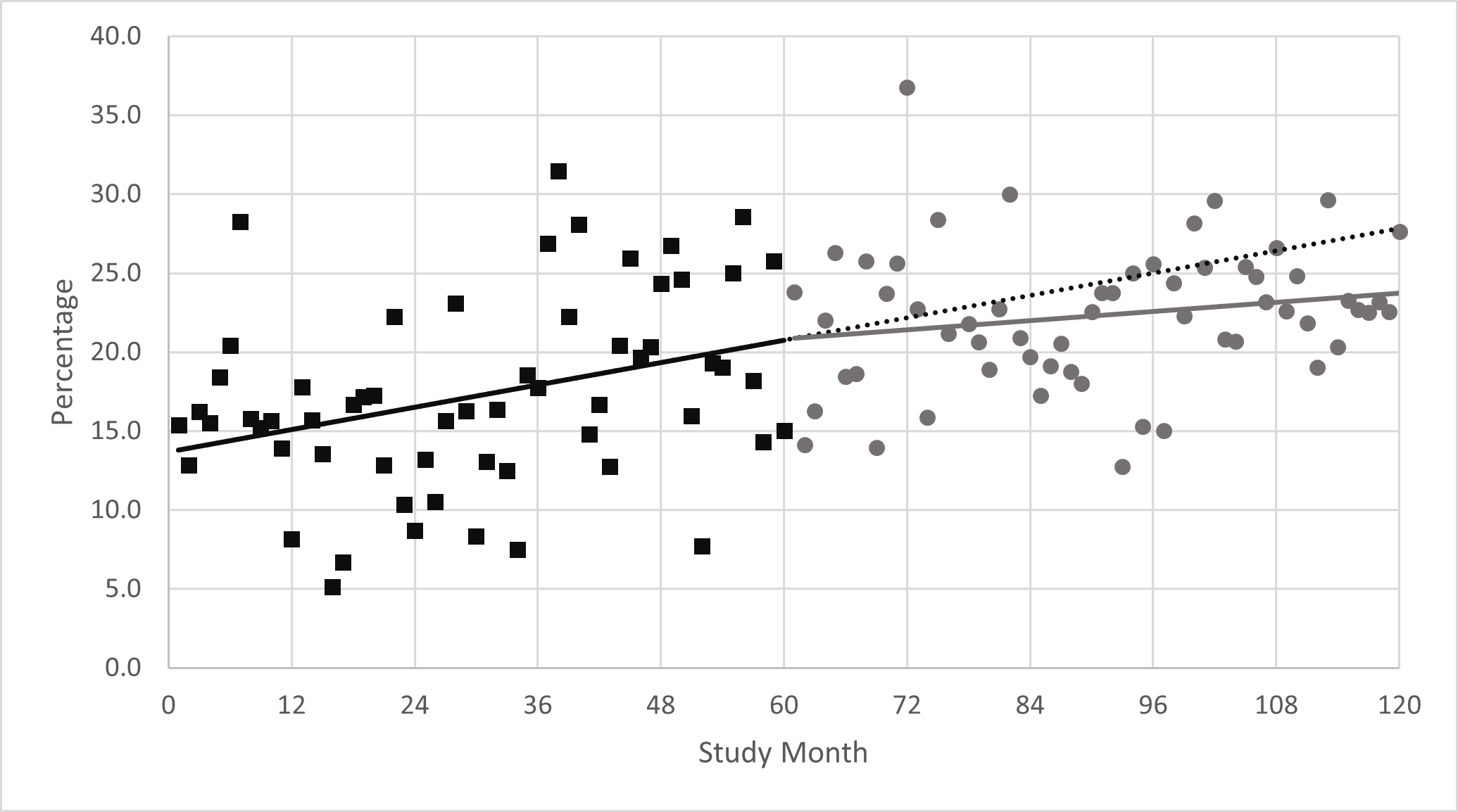

Supplement: S1 Fig — Phase 1 observed data shown as black squares, with trend shown as the solid black line. Phase 2 observed data shown as grey circles, with trend shown as the solid grey line. The Phase 1 trend is extended into Phase 2 as the dotted black line for comparison with Phase 2 observed data. Observation for study month 77 (November 2015) was identified as an outlier and excluded for this sensitivity analysis. (TIF) [file pone.0265485.s001.tif]

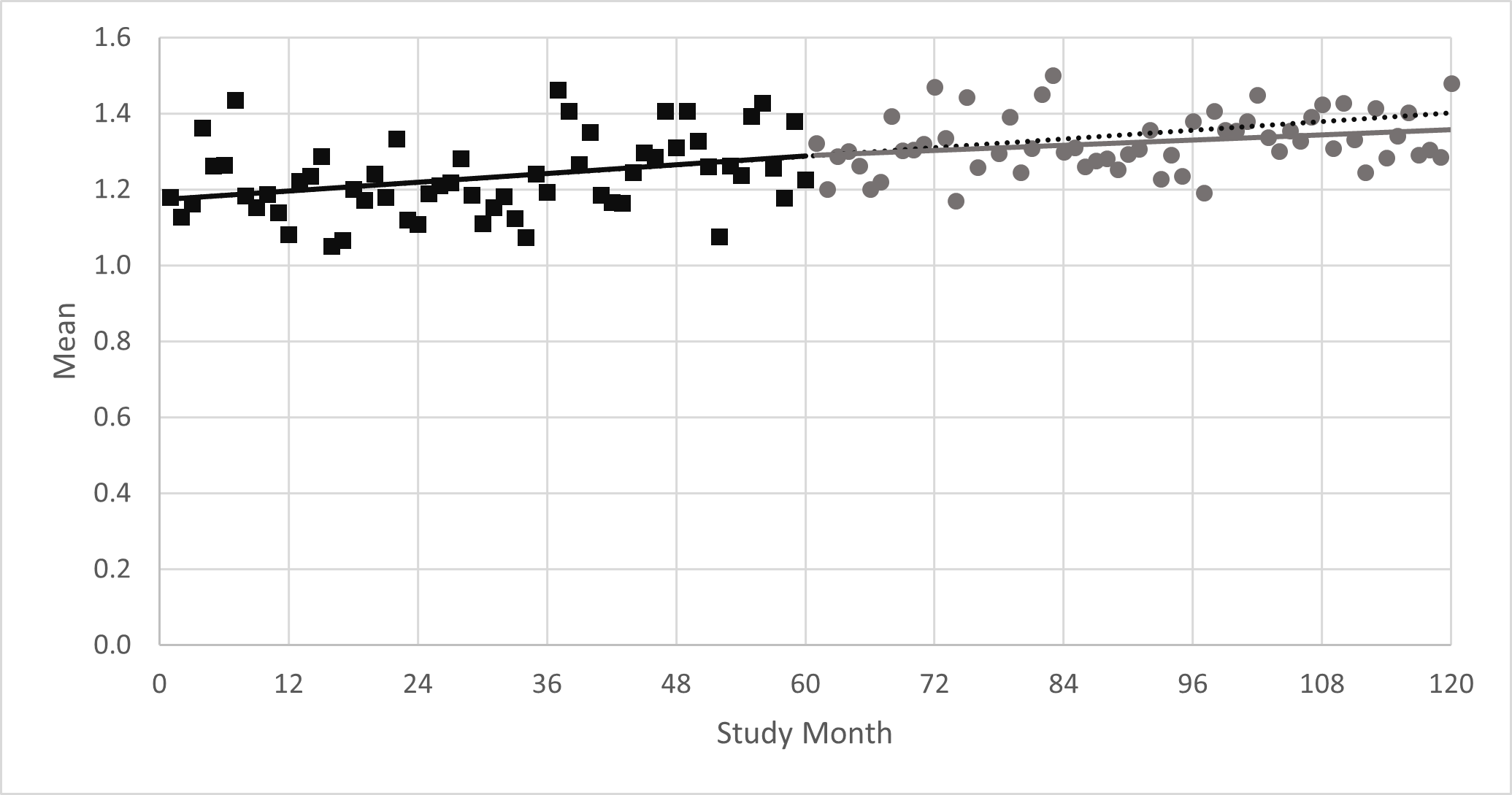

Supplement: S2 Fig — Phase 1 observed data shown as black squares, with trend shown as the solid black line. Phase 2 observed data shown as in grey circles, with trend shown as the solid grey line. The Phase 1 trend is extended into Phase 2 as the dotted black line for comparison with Phase 2 observed data. Observation for study month 77 (November 2015) was identified as outlier and excluded for this sensitivity analysis. (TIF) [file pone.0265485.s002.tif]
