# Supplementary material for: Effect of a multi-faceted rapid response system re-design on repeat calling of the rapid response team
Source: PLoS One. 2022 Mar 24;17(3):e0265485. doi: 10.1371/journal.pone.0265485 (PMC8947019; doi:10.1371/journal.pone.0265485)
Supplement: Attachment — Submitted filename: Response to Reviewers.docx [file pone.0265485.s004.docx]

Dear Dr Chen,

Thank you for the opportunity to revise our manuscript. We have addressed yours and the reviewers’ comments, both below and in the resubmission documents.

**Editor’s Comments**

1. “*Please ensure that your manuscript meets PLOS ONE's style requirements, including those for file naming*”

This has been addressed per PLOS One style templates

2. “*Upon re-submitting your revised manuscript, please upload your study’s minimal underlying data set*”

The de-identified dataset used in statistical analyses has been uploaded with agreement obtained from the Human Research Ethics Committee that approved this study

3. “*Your ethics statement should only appear in the Methods section of your manuscript*”

The ethics, funding and conflict of interest statements have been removed from the end of the manuscript per the PLOS One style template

4. “*Please review your reference list to ensure that it is complete and correct*”

This has been done, including updating reference 27 since the originally cited source is no longer available online.

**Reviewer 1 Comments**

1. “*Cite the source (reference) when models proposed by other researchers are mentioned*”

A reference is provided for ARIMA statistical modelling. The Cochrane (EPOC) reference has been updated to an original description of the segmented regression Interrupted Time Series analytical technique used.

2. “*Conclusion- Need major revision*”

The conclusion has been rewritten per your request to include the key findings and scope for future research. It now reads:

This study reports a multi-faceted RRS re-design which did not demonstrate a statistically significant effect on the percentage of patients per month having repeat calls or the number of repeat calls per admission.

The RRS re-design presented herein may be of interest to RRS clinicians and managers as a pragmatic, low-cost quality improvement initiative that could inspire future efforts to improve operational efficiency of the RRS.

3. “*Interpretation of Results: Use the reference numbers instead of author names*”

The names of the lead authors of the two cited papers have been removed.

**Reviewer 2 Comments**

1. “*I could not understand how your findings are 'suggestive of feasibility' of a redesign*”

That statement in Conclusions has been removed. The comment regarding the re-design has been rewritten to better reflect the potential contribution of the study to future research.

The conclusion now reads:

This study reports a multi-faceted RRS re-design which did not demonstrate a statistically significant effect on the percentage of patients per month having repeat calls or the number of repeat calls per admission.

The RRS re-design presented herein may be of interest to RRS clinicians and managers as a pragmatic, low-cost quality improvement initiative that could inspire future efforts to improve operational efficiency of the RRS.

2. “*Please edit the article for grammar*”

We have thoroughly perused the manuscript and used Microsoft Word grammar checks. Errors have been corrected per PLOS One style and language requirements.

**Reviewer 3 Comments**

None to address
