# Supplementary material for: Effect of a multi-faceted rapid response system re-design on repeat calling of the rapid response team
Source: PLoS One. 2022 Mar 24;17(3):e0265485. doi: 10.1371/journal.pone.0265485 (PMC8947019; doi:10.1371/journal.pone.0265485)
Supplement: Attachment — Submitted filename: Response to Reviewers Comments.docx [file pone.0265485.s005.docx]

Dear Dr Chen,

Thank you for the opportunity to revise our manuscript again. We have addressed the reviewers’ comments, both below and in the resubmission documents.

**For the Editor**

Further amendments have been made to the Discussion section of the manuscript. These should add clarity to some of the points raised by the Reviewers and better detail the relevance of this study as a component of the *Impact of Non-Technical Skills on Performance and Effectiveness of a Medical Emergency Team* research project.

These edits and those in response to Reviewer 1 (as detailed below) are shown as Track Changes within the re-submission documents.

**Reviewer 1 Comments**

1. “*Conclusion: Need revision. A conclusion should address: answers research questions/objectives; explains discrepancies and unexpected findings; states importance of discoveries and future implications.*”

The conclusions of both the abstract and manuscript have been thoroughly revised and rewritten per your request to clarify the study objectives, key findings, discrepancies and the scope for iterative research and quality improvement.

The abstract conclusion now reads:

“This study of an RRS re-design showed modest, but not statistically significant, reductions in the proportion of admissions with repeat calls and the mean number of repeat calls per admission. Given the economic and workforce capacity issues that all health care systems now face, even small improvements in the RRS may have lasting impact across the organisation. For the potential interest of RRS managers, this paper presents a pragmatic, low-cost initiative intended to enhance communication and cooperation at RRT calls.”

The manuscript conclusion now reads:

“This study reports a multi-faceted RRS re-design which was associated with a modest, but not statistically significant, reduction in the percentage of patients per month having repeat calls and the average number of repeat calls per admission.

In an era of economic and health workforce constraints, even small potential improvements may still have relevance to organisations. This RRS re-design (and assessment thereof) has scope for further refinement, and may be of interest to RRS clinicians and managers seeking to implement their own pragmatic, low-cost quality improvement initiatives.”

**Reviewer 2 Comments**

None to address
